# Timing of decompressive craniectomy and short-term outcomes in pediatric severe traumatic brain injury: a nationwide observational study in Germany

**DOI:** 10.1038/s41598-026-35837-3

**Published:** 2026-01-17

**Authors:** Rayan Hojeij, Pia Brensing, Bernd Kowall, Andreas Stang, Michael Nonnemacher, Ursula Felderhoff-Müser, Philipp Dammann, Marcel Dudda, Christian Dohna-Schwake, Nora Bruns

**Affiliations:** 1 Department of Pediatrics I, Neonatology, Pediatric Intensive Care Medicine, Pediatric Neurology, and Pediatric Infectious Diseases, University Hospital Essen, University of Duisburg-Essen, Essen, Germany; 2 C-TNBS, Centre for Translational Neuro- and Behavioural Sciences, University Hospital Essen, University of Duisburg-Essen, Essen, Germany; 3 Institute for Medical Informatics, Biometry and Epidemiology, University Hospital Essen, University of Duisburg-Essen, Essen, Germany; 4 Department of Neurosurgery and Spine Surgery, University Hospital Essen, University of Duisburg-Essen, Essen, Germany; 5 Department of Trauma, Hand, and Reconstructive Surgery, University Hospital Essen, Essen, Germany

**Keywords:** Decompressive craniectomy, Mortality, Pediatrics, Severe head injury, Timing, Diseases, Medical research, Neurology, Neuroscience

## Abstract

Decompressive craniectomy (DC) is a critical intervention for managing severe traumatic brain injury (sTBI) in children when medical therapy fails, but the optimal timing remains unclear. This study evaluated the association between different DC timing and short-term outcomes in pediatric sTBI in Germany. A retrospective cohort study of the German national hospital discharge database was conducted for cases < 18 years undergoing DC following sTBI were extracted from 2016 to 2022. Time from admission to DC were calculated as complete hours and data were compared between early (time to DC ≤ 2 h) and late DC (> 2 h). Hierarchical logistic regression models evaluated the association of DC timing with in-hospital mortality, functional outcomes (Pediatric Complex Chronic Conditions (PCCC) ≥ 2)), poor outcome (composite outcome of death or PCCC ≥ 2), length of hospital stay, days on mechanical ventilation (MV) and coding of seizures. Among 13,492,821 children hospitalized, 9,495 had sTBI. DC was performed in 598 cases and half of surgeries were performed within the first two hours after admission. Higher odds of death (adjusted odds ratio [OR] 2.89; 95% confidence interval [95%CI] 1.43–5.85) were observed in the early versus late DC groups. However, in survivors, early DC was linked to shorter durations MV and hospital stay. Sensitivity analyses across multiple thresholds of DC timing confirmed mortality and MV findings. Early DC in pediatric sTBI was primarily performed as an urgent intervention in critical injured patients, yet survivors showed faster recovery with few MV days and hospital stay.

**Supplementary Information:**

The online version contains supplementary material available at 10.1038/s41598-026-35837-3.

## Introduction

Decompressive craniectomy (DC) has emerged as a rescue measure to relieve refractory intracranial hypertension and prevent secondary cerebral ischemia or herniation in children with severe traumatic brain injury (sTBI). Primary DC, performed in the initial phase when swelling occurs or is anticipated, is often carried out concurrently with hematoma evacuation following severe head trauma^1^. On the other hand, secondary DC serves as a last-tier option to treat refractory elevation of intracranial pressure (ICP) despite medical management (MM) during the clinical course^2^.

Very few randomized controlled trials (RCTs) have evaluated the effect of DC on outcomes in adults and children with persistent ICP elevation. The only pediatric RCT, conducted in 2001 with 27 children, found that DC performed at a median time of 17 h after admission was associated with good outcomes and significant ICP reduction compared the MM group^3^. In adults, the DECRA trial included patients with severe diffuse traumatic brain injury and ICP elevation above 20 mmHg for more than 15 min within the first 72 h after injury. Patients were randomized to either bifronto-temporo-parietal DC or standard care^4,5^. Early DC was associated with poorer functional outcomes at 6 months of follow-up^5^, and higher rates of vegetative state at 12 months among survivors^4^. The RESCUEicp trial included patients ≥ 10 years, were randomized to undergo DC (as a last tier intervention) after optimized MM or continued medical care if ICP remained above 25 mmHg for 1 to 12 h^6^. Six-month outcomes yielded lower lethality but higher frequencies of vegetative state than the medical care group. Due to these inconsistent trials regarding timing of decompression, recommendations for DC remain rather imprecise, leaving the decision to decompress at the discretion of the treating medical team.

Although a brief correspondence has previously addressed case fatality in this population^7^, the broader relationship between timing of DC and clinical outcomes remains undefined. Observational studies have produced varying results when comparing early to late DC: some suggesting early DC reduce morbidity-mortality^8,9^, while others found no association^10^. Despite these contradictory findings on the general relationship between timing and outcomes, the literature lacks a universal definition for “early” or “late” DC, as studies used varying cut-off times to define groups, ranging from 2 to 24 h. Further, the timing of DC is influenced by factors such as injury severity, neurological status, individual patient characteristics, health care system infrastructure, and the neurosurgeon’s decision.

The aim of this study was to explore the association between timing of DC and outcomes in the German hospital dataset (GHD) in pediatric cases with sTBI. This manuscript presents a comprehensive analysis, including data on primary and secondary short term outcomes that builds up our earlier correspondence.

## Materials and methods

### Study design and setting

We conducted a retrospective cohort study using patient data from the GHD to investigate the association between timing of DC and short term outcomes in pediatric cases with sTBI. The comprehensive nature of the study and dataset allows an exhaustive analysis of a large dataset, encompassing the entire public hospitals across Germany.

German hospitals have been reimbursed based on diagnosis related groups (DRGs) since 2004. According to Section 21 of the German Hospital Remuneration Act (KHEntgG), German hospitals are required by law to share data on all hospital admissions with the hospital payment system (InEK). After passing plausibility checks, these data are irreversibly anonymized and transmitted to the Federal Statistical Office (FSO), where they are made available for scientific research through the Research Data Centre (Forschungdatenzentrum, FDZ). As these hospitalization data are mandatory for reimbursement, hospitals are strongly incentivized to provide complete data sets.

The present study was conducted exclusively using these fully anonymized administrative data. Under German law, such data do not constitute research involving human participants; therefore, ethical committee approval was not required, and the requirement for informed consent was waived. Access to the data was granted only after formal approval by the Research Data Centre’s access committee. All analysis and outputs are reviewed and endorsed by the federal data protection officer and comply with applicable federal data protection regulations. Detailed information on the structure of the DRG dataset and the legal and ethical framework governing its use is available at the Research Data Centre for Health (https://www.forschungsdatenzentrum.de/en/health/drg) and in the terms of use (https://www.forschungsdatenzentrum.de/en/terms-use).

### Case selection

Inclusion criteria were cases < 18 years of age discharged from public hospitals in Germany between 2016 and 2022 with sTBI who underwent DC. Selected cases had TBI as primary discharge diagnosis (ICD-10 code: S06) identified via codes of the International-Classification of Disease, 10th edition, German modification (ICD-10-GM). We defined sTBI as an Abbreviated Injury Scale (AIS) of the head ≥ 3. Patients who underwent DC were identified and selected via operation and procedure (OPS) codes (5-0120, 5-0101, 5-0104). Patients were followed-up until discharge or death from the hospital.

### Data extraction

We extracted data from the GHD, including patient demographics, clinical variables (type of injury, complications), survival status at discharge, length of stay (LOS), and procedures details (DC, ICP monitoring, EVD). Time and date of admission, discharge, surgery for DC and ICP monitoring were obtained from the database and time from admission until surgery/placement of ICP monitor or EVD were computed. Time was calculated in completed hours, rounding to the nearest full hour to minimize classification bias around the threshold.

The following type of head injury codes were extracted: traumatic cerebral oedema (ICD-10-GM: S06.1), traumatic subdural hemorrhage SH (ICD-10-GM: S06.5), traumatic epidural hemorrhage EH (ICD-10-GM: S06.4) and traumatic subarachnoid hemorrhage SAH (ICD-10-GM: S06.6).

Injury severity was quantified as previously described based on the AIS, injury severity score (ISS) and using a validated ICD based injury severity score (ICISS)^11^. These scores were used to categorize injuries and to adjust for injury severity. The ICISS is empirically derived by calculating survival proportions (or survival risk ratios SRR) for each injury diagnosis code. The ICISS was used to estimate injury severity using SRR. A lower SRR value indicates higher risk of death Single-ICISS was extracted as the single worst injury (= lowest value) from all trauma-related ICD codes for each case, while the multiplicative ICISS accounted for the multiple injuries by multiplying the assigned values of all trauma-related ICD codes of each case.

Functional outcome at discharge was assessed using the Pediatric Complex Chronic Conditions (PCCC) Classification with minor modifications that were necessary due to the inherent structure and the lack of the long-term follow-up data of the GHD^12^ (See Supplementary Table 1, Additional File 1). The PCCC was designed to identify and quantify conditions in children that are likely to persist for at least one year. A score is assigned for each case a score ranging from 0 to 12, reflecting the total number of chronic conditions present. While PCCC is not a direct neurological functional score, it was adopted due to feasibility in administrative data and has been use in other pediatric outcome studies.

Organ dysfunction was assessed and quantified by summing up extracted binary scores that indicate organ dysfunction (See Supplementary Table 2, Additional File 2: Supplemetary Table).

### Timing of surgery

We calculated time from admission to DC was rounded to completed hours and categorized cases into two groups: early time to DC (within the first 2 completed hours after admission) and late time to DC (performed more than 2 h post admission). This categorization was primarily data-driven, as there is no widely accepted definition of early versus late DC. The cutoff at 2 completed hours was based on the cohort´s median time to DC, reflecting the actual distribution of DC timing in our specific patient population^7^. To address potential misclassification, sensitivity analyses were conducted using alternative time thresholds of 1, 3, 12, and 24 h to test the robustness of the results.

### Outcomes measures

The primary outcome was in-hospital death following DC. Secondary outcomes included a poor functional outcome, defined as PCCC ≥ 2, proposed by Simon et al. as significant chronic conditions affecting body systems that are expected to last at least a year^13^. Other secondary outcomes were LOS, duration of MV in days, coding of seizures, and poor outcome, defined as a composite outcome of death or PCCC ≥ 2.

### Missing data

There were no missing data on age, main diagnosis, LOS, or survival at discharge. In our analyses, we had to assume that an ICD-code or OPS code that was not documented meant that the diagnosis was not present, or the procedures was not done, respectively.

We nevertheless assume that extensively reimbursed procedures such as DC are carefully coded, which justifies our selection of these codes. In addition, 9 patients with missing information on time or date of admission, discharge or DC were removed from the final analysis.

### Statistical analysis

Descriptive statistics were used to summarize demographic and clinical characteristics within each group. Continuous variables were described using median and interquartiles (Q1-Q3), while count data were presented as frequencies and percentages.

**Fig. 1:**
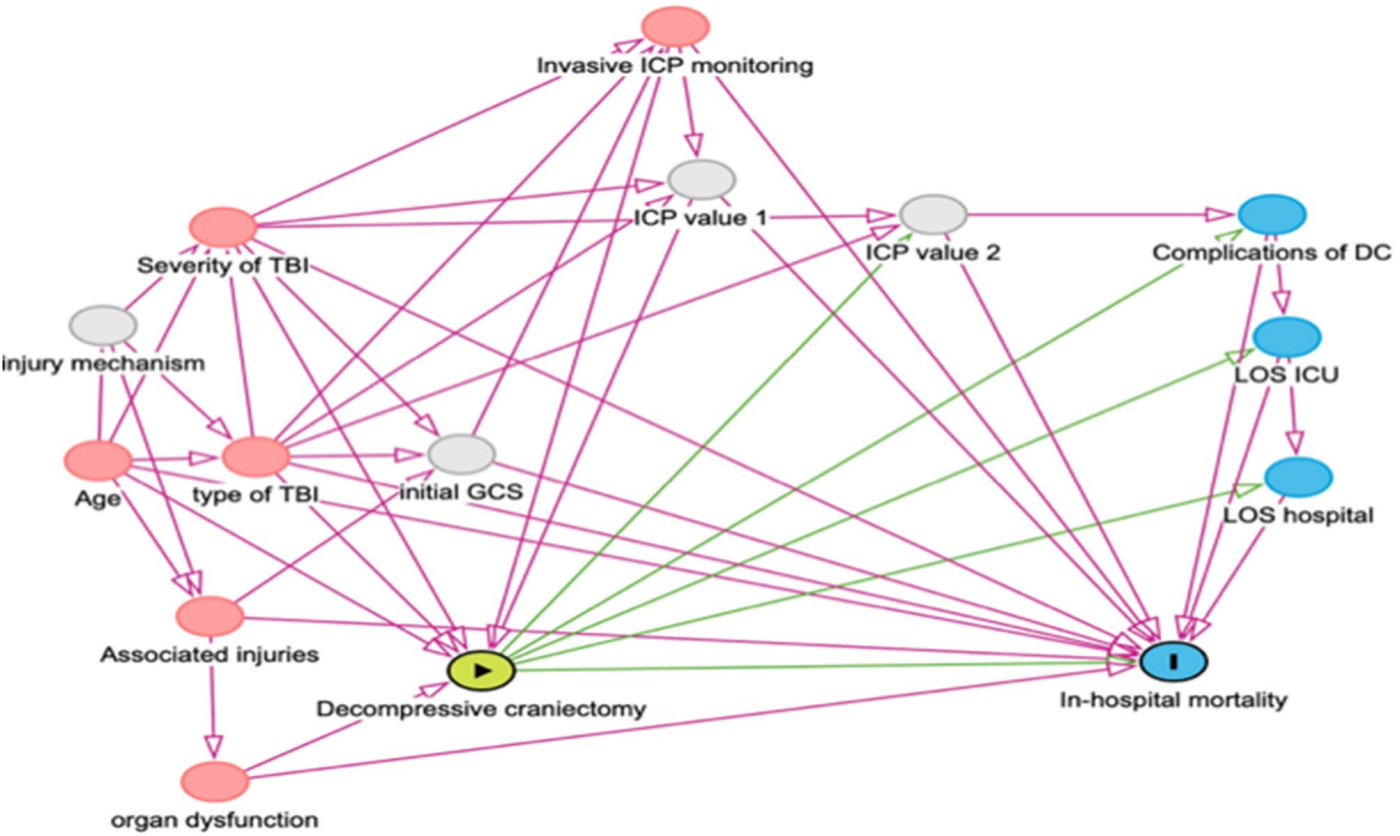
Directed acyclic graph to identify the minimally sufficient adjustment set for multivariable analyses. Yellow circle: exposure of interest; blue circle with “I”: outcome; grey circles: covariates not available/unmeasured. Minimally sufficient adjustment set: age, type of TBI, Associated injuries, organ dysfunction, invasive ICP monitoring and injury severity.

Characteristics associated with time to DC and primary outcome (in-hospital mortality) were selected as potential confounders based on theoretical considerations using directed acyclic graphs (DAGs)^14,15^, a method recommended for empirical pediatric and critical care research^16,17^ (Fig. 1). The covariates -age, sex, type of head injury, coma, severity of the injury (ICISS), ICP and organ dysfunction- were chosen as the minimal set needed for adjustment in the DAG. The severity of the injury was accounted for by using the single ICISS, where the worst single injury may predict mortality in cases of sTBI.

Hierarchical logistic regression models were used to estimate the odds of outcomes associated with the timing of the intervention (early versus late DC), while accounting for the clustering of cases within centres (identified via the institutional identifier)^18,19^. To account for clustering, institutional identifiers were included as a random effect in the model. Adjusted odds ratios were calculated after controlling for the selected confounders, determined using the minimal adjustment set required by DAGs (Fig. 1). Adjusted least square means were computed to estimate and compare the adjusted group means of MV and LOS in survivors while accounting for covariates included in the model. All calculations and analyses were performed using SAS release 9.4 and SAS Enterprise Guide 7.3 (SAS Institute, Cary, North Carolina, USA).

### Sensitivity analyses

Sensitivity analyses were conducted to assess the robustness of our findings, reclassification of early and late DC using alternative thresholds (≤ 1 h ,≤ 3 h, ≤ 12 h and ≤ 24 h) to surgery after admission. Directional consistency of finding across these thresholds was evaluated to assess the robustness of results.

### Ethics approval and consent to participate

Only secondary anonymized data were used. Access to data is possible after inquiring and signing a data confidentiality agreement with the Federal Statistical Office. All results have been endorsed by the federal data protection officer. The present study was conducted exclusively using these fully anonymized administrative data. Under German law, such data do not constitute research involving human participants; therefore, ethical committee approval was not required, and the requirement for informed consent was waived. The legal and ethical framework governing the use of these data is publicly available at https://www.forschungsdatenzentrum.de/en/terms-use .

## Results

### Patient characteristics

**Fig. 2:**
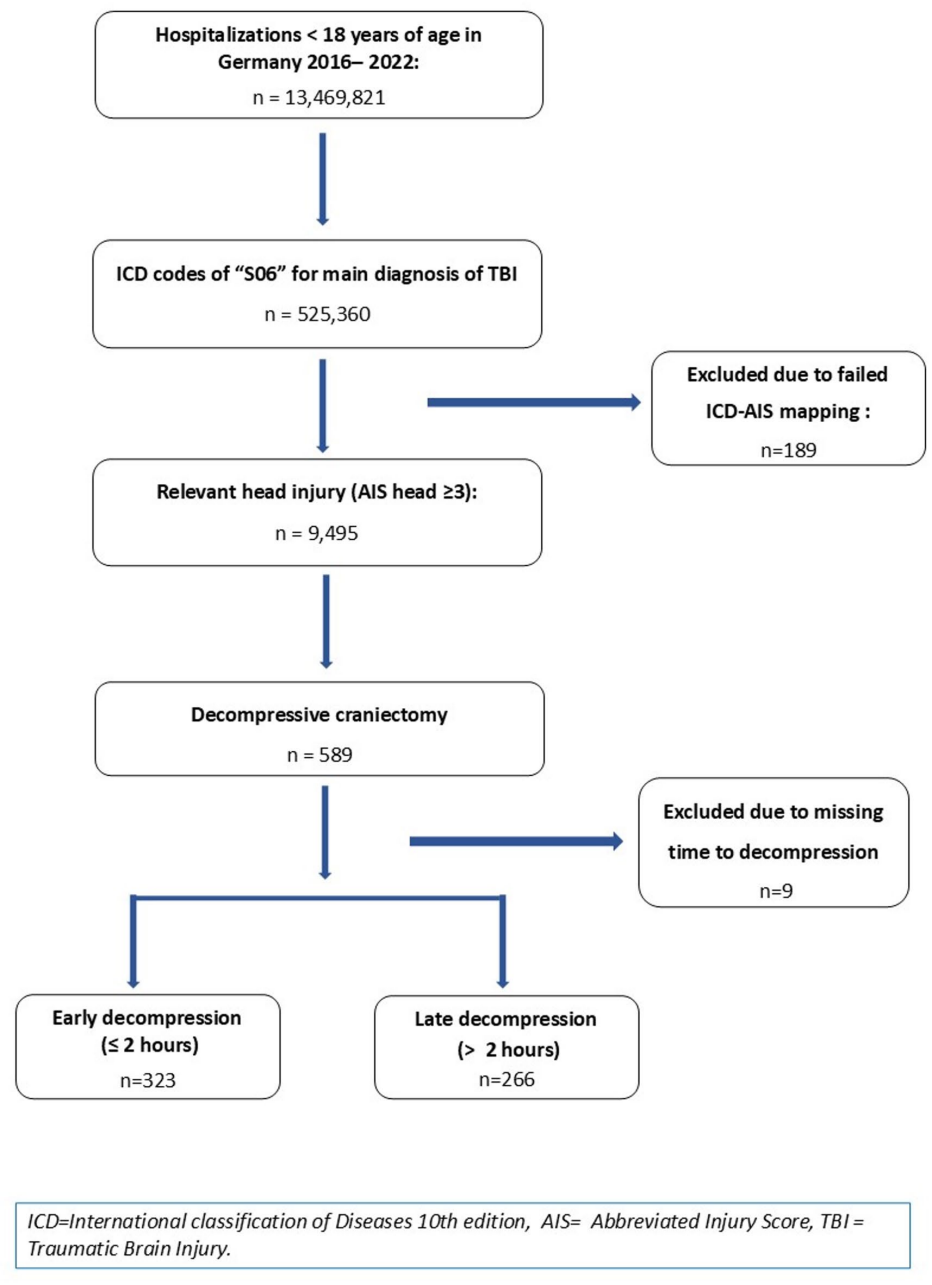
: Flowchart of included traumatic brain injury cases aged 0–17 years in Germany, 2016–2022.

Between 2016 and 2022, a total of 13,469,821 pediatric cases were discharged from public hospitals across Germany. Among these cases, 525,360 received a primary discharge diagnosis of TBI, 9,495 of which were severe TBI cases with AIS head ≥ 3. 589 (6.3%) cases underwent DC during their hospital stay, 323 (54.8%) within the first two hours and 443 (75.2%) within the first 24 h of hospitalization (Fig. 2).

The cases that received DC were predominantly male (67.1%) with a median age of 12 years (IQR 4–16) and a median hospital stay of 17 days. 27% of DC cases died in hospital with a median time from admission to death of 2 days. The majority of DC cases involved brain edema and subdural hemorrhage, affecting (382/589) 64.9% and (411/589) 69.8% of patients, reported as separate findings respectively. One third of the cases were in coma 31.2%, and more than half received invasive ICP monitoring (51.8%).

### Differences by timing

Characteristics of patients receiving early, and late DC were largely similar (Table 1). Differences were observed for lethality (37.5% in early DC vs. 15.8% in late DC) and hematoma evacuation (72.0% vs. 52.6%). A shorter median hospital stay of 11 days was observed in the early DC group compared to 22 days after late DC. While half of the cases received ICP monitoring in both the early and late DC group, 77.9% of the ICP monitoring in the late DC group was performed before DC. The median time from admission to ICP monitoring in the early DC group was 67 min, with 86% of ICP monitors being inserted during the same surgery in which the DC was performed. Similarly, we observed that 58.6% of those who received EVD were performed before the DC surgery in the late DC group, where 57.1% in the early DC group had EVD intraoperatively.

### Differences by timing in survivors

In survivors (*n* = 426), a shorter median LOS was observed in the early DC group, with 19 days (IQR 11–33) compared to 24 days (IQR 16–35) in the late DC group. Early DC patients also required fewer days of mechanical ventilation, with a median of 5 days (IQR 1.7–12) versus 10.8 days (IQR 5.9–19) after late DC. Tracheostomy and gastrostomy were more frequently performed in the late DC group (33% vs. 21% and 19% vs. 13%, respectively). No other clinically relevant differences between the groups were observed (Table 1). PCCC category results are presented in Supplementary Fig. 1, Additional File 1). Higher proportions of respiratory (29.6% vs. 26.3%), technical dependency (18.8% vs. 11.3%), and gastroenterological (7.5% vs. 4.3%) categories were observed in the late DC group compared with the early DC group.

### Patient outcomes

**Fig. 3:**
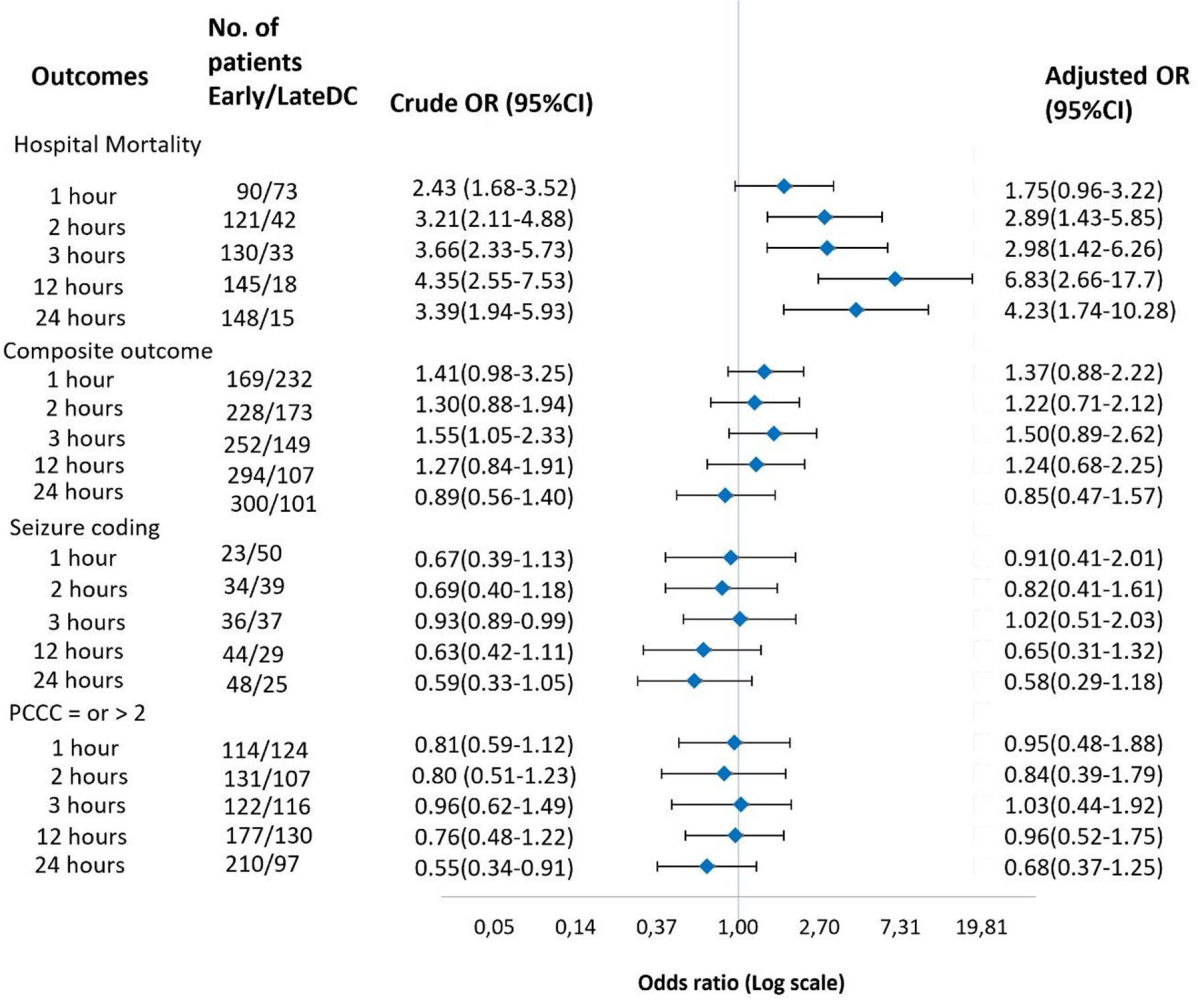
Crude and adjusted odds ratios of adverse outcomes comparing early versus late decompressive craniectomy in children with severe traumatic brain injury in Germany 2016–2022 using multiple timing to DC thresholds cutoffs. Composite outcome = in-hospital death or PCCC ≥ 2. Mortality and composite outcomes were assessed in the full cohort (*n* = 589), PCCC and seizure outcomes were evaluated in survivors only (*n* = 426). All models were adjusted for age, sex, single ICISS, coma, POFI, ICP monitoring and type of injury.

Logistic regression showed higher adjusted odds for death (odds ratio (OR) 2.89 (95% CI: 1.43–5.85) in early versus late DC groups (Fig. 3). We failed to find a significant associations between early and late DC with respect to composite outcome (Fig. 3) .

### Survivor’s outcomes

**Fig. 4:**
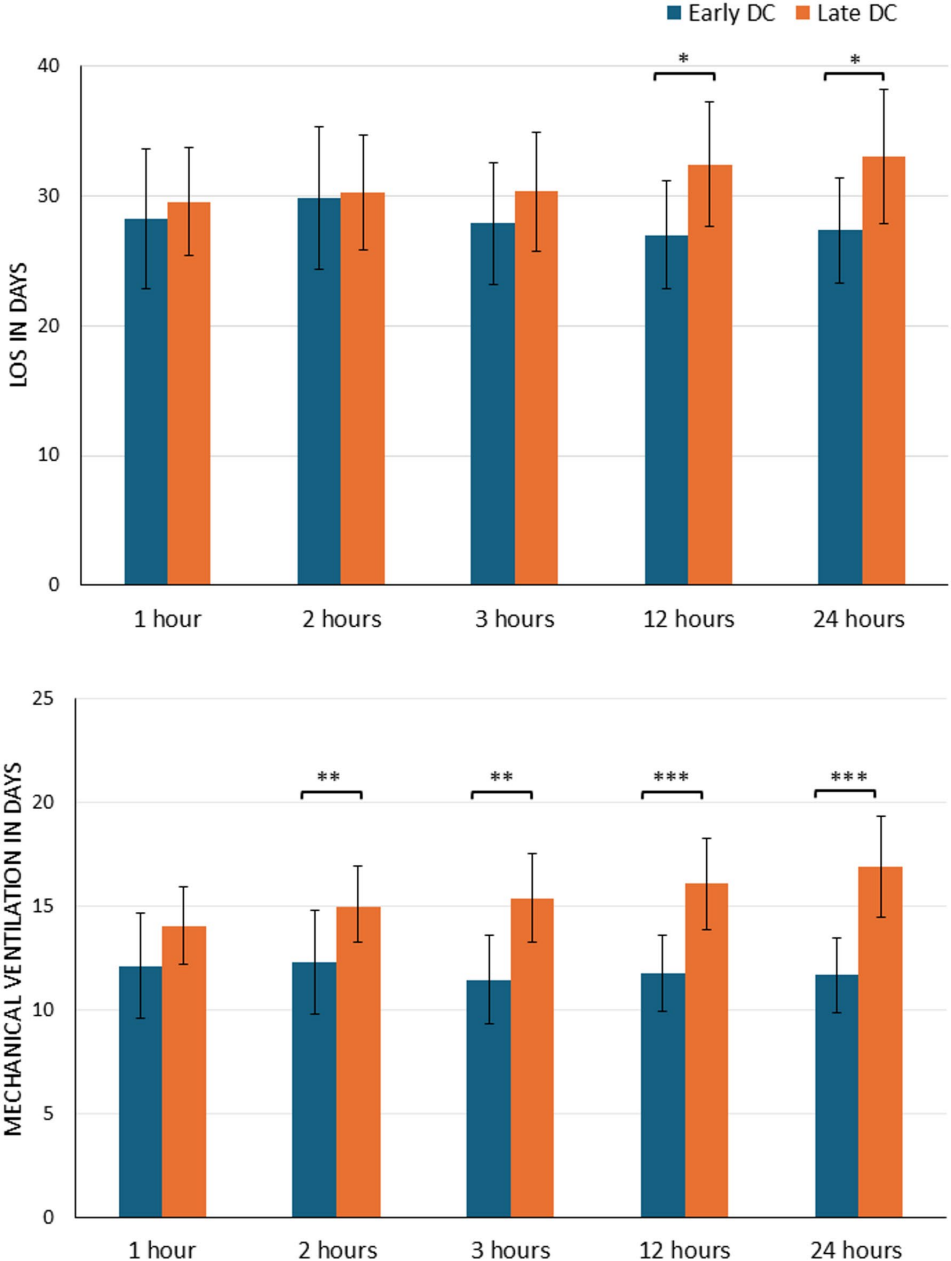
Adjusted least square means of mechanical ventilation in days, and length of stay by early versus late DC survivors’ cases using different timing cut-offs thresholds. Adjusted model controlled for age, sex, single ICISS, coma, ICP monitoring, POFI and type of injury. Vertical bars denote 95% confidence intervals. *p < 0.05 **p < 0.01 ***p < 0.001.

We failed to find association between early/late DC and PCCC ≥ 2 or seizure coding (Fig. 3). However, the adjusted mean duration of MV was significantly higher in the late DC group (15.0 days; 95% CI: 13.0–17.0) compared with the early DC group (11.5 days; 95% CI: 9.4–13.5; *p* = 0.002) (Fig. 4).

### Sensitivity analysis

The sensitivity analysis showed higher odds of death with an aOR of 2.98 (95% CI: 1.42–6.26) in the early (≤ 3 h to DC after admission) versus late (> 3 h) DC groups. Similarly, higher odds of death were observed when using the 12-hour (aOR 6.83, 95% CI: 2.66–17.7) and 24-hour (aOR 4.23, 95% CI: 1.74–10.28) cut-offs. The association between the duration of MV and time to DC in survivors became stronger across all thresholds, and the results remained consistent regardless of the early or late DC definition except for the 1 h cut-off threshold. However, differences in hospital stay between groups were only observed at the 12- and 24-hour cut-offs. At the 12-hour cut-off, survivors in the late DC group had longer hospital stays, with a mean LOS (95% CI) of 32.5 days (28.0–37.3; *p* = 0.03) versus 27.0 days (22.0–31.2), in the early DC group, and 33.1 days (28.8–37.4; *p* = 0.03) versus 27.4 days (23.3–31.4) at the 24-hour cut-off. No such differences were observed at the 1-, 2-, or 3-hour thresholds. No other significant differences in outcomes were found across the various DC timing cut-offs.

## Discussion

### Key findings

In this nationwide retrospective cohort study of pediatrics TBI in Germany, earlier DC was associated with higher in-hospital mortality. Among survivors, earlier DC was linked to less days on MV duration and hospital stays, while we failed to find differences in functional outcomes between groups.

This is the first large-scale, nationwide analysis evaluating the timing of DC in children and its association with outcomes. Most DCs were performed on the first day of admission, with half occurring within the first two hours of hospital arrival. Higher rates of tracheostomy and gastrostomy placement were observed in the late DC group survivors, reflecting greater neurological impairment and the need for prolonged airway and nutritional support.

### Clinical implications

In our study, timing of DC appears closely linked to injury severity and clinical urgency at admission. Early DC was frequently performed in parallel with mass lesion evacuation and was rarely preceded by ICP monitoring, suggesting that the decision for surgery was driven by immediate neurosurgical necessity rather than escalating ICP. Children undergoing early DC also showed higher rates of severe extracranial injuries, which likely contributed to the increased in-hospital mortality observed in this group. These findings align with previous studies reporting that primary DC is more often applied in the most critically injured patients, particularly those with low Glasgow Coma Scale scores, thicker hematomas, larger midline shifts, and polytrauma^20–23^.

Compared with early DC cases where urgent DC was prioritized, late DC was more frequently preceded by ICP monitoring and EVD use, reflecting a stepwise escalation of care in accordance with tier-based TBI management guidelines. Although international guidelines recommend ICP monitoring to manage ICP levels in sTBI, only half of the patients in our study received continuous ICP evaluation, particularly important in the late DC cases, where elevated ICP can prompt decision to DC^24–26^. Among DC cases who underwent ICP monitoring preceding DC, 115 out of 127 were monitored within the first 24 h of admission, of which 19 cases (16.5%) died. While there is no class I evidence supporting ICP monitoring after primary DC, several adults retrospective studies concluded its usefulness in guiding postoperative therapy and reduce in-hospital mortality, especially in the presence of brain swelling^27–29^.

Among survivors, earlier DC was associated with fewer days of MV and shorter LOS, despite no differences in functional outcome at discharge. This suggests that early DC may accelerate physiological stabilization without substantially altering early neurological status, which is likely determined by the severity of the initial brain injury. Long-term follow-up is needed to determine whether these advantages translate into lasting neurological improvement.

Early DC may be lifesaving in selected cases of rapid neurological deterioration, and survivors may experience a faster acute recovery. In contrast, late DC survivors is more often performed after evidence-based escalation of invasive therapies and may lead to prolonged survival with complex post-acute care needs, including longer ventilatory, nutritional support and hospital stay.

### Comparison to previous studies

Previous RCTs, such as the DECRA conducted in Australia/New Zealand between 2002 and 2010 and included 155 adults, and RESCUEicp trials (UK, Europe, 2004–2014; 408 adults), have evaluated outcomes after secondary DC in adults. In children, evidence is limited to one RCT, case series, and retrospective studies, many of which had small sample sizes, were single-center, and compared DC to medical management and had higher risk of selection bias^3,8,10,30–35^.

Only a few retrospective pediatric TBI studies have investigated the timing effect of DC, with varying definitions of early and late procedures^10,32,36^. This variability in timing, along with heterogeneous patient characteristics across studies, makes direct comparisons challenging. In our nationwide German cohort, early DC was associated with higher lethality and shorter time to death, with a marked percentage of children undergoing hematoma evacuation during the same surgery. Conversely, the DECRA trial defined early DC as occurring within 38 h and refractory to first-tier treatment, excluded patients with intracranial hemorrhage, reporting equal mortality rates between early DC and MM^5^. In the RESCUEicp study, where DC was considered a last-tier therapy (median randomization time: 44.3 h), demonstrated lower mortality rates.

A recent multicenter retrospective study conducted by Nagy et al. (2021, United States) included 78 children with sTBI, also found that the acute intervention group in children (< 24 h of admission) had higher mortality compared to the subacute group (≥ 24 h) with no differences in functional outcomes (33.3% vs. 18.2%, respectively)^10^. However, multivariable analysis and thus adjustment for confounders were not performed due to the limited sample size. Similarly, another retrospective study conducted in Honeybul et al. (2010, Australia) analyzed 37 pediatric cases, evaluated the association between early DC (time from injury to surgery < 12 h) and 14 days in-hospital mortality found that children with higher ISS had increased odds of death, however there were no comparison to a late DC group^33^. In adults, Cooper et al. (2004, Australia) and Timofeev et al. (2006, UK) both conducted retrospective analyses (each with 100–150 patients), have evaluated the association between timing and outcomes (defining early surgery as 3- and 4-hours from injury to surgery respectively) and showed no differences in mortality compared to the late DC group^37,38^. Our dataset represents the first nationwide German pediatric cohort rather than single or few centre data, including broader cases of DC in pediatrics following sTBI and more diverse practices. To address the heterogeneity in timing definitions, we conducted sensitivity analyses using 1-, 3-, 12-, and 24-hour cut-offs. These thresholds were chosen based on the distribution of our data, where most DC procedures occurred within the first few hours after hospital admission, and on previously established thresholds reported in the literature.

### Strengths and limitations

The present study has several limitations, including lack of information on clinical reasoning for DC timing, physiological parameters from the shock room and ICU, ICP measurements, and medical management before and after surgery. Additionally, time-related information such as the duration from the accident to hospital admission, and clinical information of neurological status (Glasgow coma scale, Marshall/Rotterdam scores) were not available.

There is potential for confounding by indication related to injury severity and the factors leading to hematoma evacuation with subsequent DC, as documentation on these factors may be lacking. However, we attempted to adjust for available confounders to the best of our ability, recognizing that often a subset of confounders can effectively mitigate much of the residual confounding. Most DCs in our cohort were performed within the first 48 h after admission (more than 85%) ; however, a few procedures occurred several days later, reflecting heterogeneous clinical circumstances that may have influenced outcomes.

Future research should include prospective data collection and standardized criteria for surgical decision-making to optimize DC timing in pediatric sTBI. Moreover, prospective studies evaluating the role and benefits of ICP monitoring in this population would help clarify its risk–benefit profile. Despite these limitations, this study comprises a considerable number of pediatric DC cases, providing a comprehensive overview of DC timing and outcomes after sTBI in Germany. While the findings are most applicable to similar healthcare settings, future research should explore their generalizability to broader populations.

## Conclusion

This nationwide study of DC after severe pediatric TBI in Germany found that early DC, often necessary to evacuate a massive lesion, was most likely conducted as a primary-rescue intervention compared to late DC. Despite this, survivors following earlier DC required fewer days on MV and spent less days in the hospital suggesting faster recovery and discharge. Future research should focus on further developing timeline guidelines and incorporating standardized decision criteria to improve patient outcomes. A comprehensive risk assessment should guide clinical decision-making, weighing the potential long-term complications against this strategy. Potential complications, such as infections, cerebrospinal fluid leaks, and the need for re-surgery, must be discussed with parents to ensure informed decision-making regarding treatment strategy.

**Table 1:** Children’s characteristics stratified by early (time to DC ≤ 2 h) and late DC (> 2 h) after hospital admission for severe traumatic brain injury in Germany, 2016–2022.

	Total (*N* = 589), No.(%)	Early DC (*n* = 323), No.(%)	Late DC (*n* = 266), No. (%)
Age, median (IQR)	12 (4–16)	12 (3–16)	11 (5–16)
Age categories			
0–5	171 (29.0)	96 (29.7)	75 (28.2)
6–12	121 (20.5)	60 (18.6)	61 (22.9)
13–17	297 (50.4)	167 (51.7)	130 (48.9)
Sex, male	395 (67.1)	220 (68.1)	175 (65.8)
LOS in days, median (IQR)	17 (5–29)	11 (2–23)	21.5 (9–32)
LOS in survivors, median (IQR)**	22 (13–35)	19 (11–33)	24 (16–35)
Transferred cases	50 (8.4)	27 (8.3)	23 (8.6)
In-hospital mortality	163 (27.7)	121 (37.5)	42 (15.8)
14-days In-hospital mortality,	155 (26.3)	116 (35.9)	39 (14.7)
Time to death after DC, median (IQR)	2 (1–6)	2 (1–4)	4 (1–5)
Mechanical ventilation (days), median (IQR)**	7.5 (2.5–15.8)	5 (1.7–12)	10.8 (5.9–19)
Serious extracranial injuries (AIS ≥ 3)			
Thorax	195 (33.1)	118 (36.5)	77 (28.9)
Abdomen	28 (4.7)	21 (6.5)	7(2.6)
Spine	11 (1.9)	6 (1.9)	5 (1.8)
Type of head injury			
EH (Epidural)	166 (28.2)	92 (28.5)	74 (27.8)
SH (Subdural)	411 (69.8)	231 (71.5)	180 (67.7)
SAH (Subarachnoid)	223 (37.9)	116 (35.9)	107 (40.2)
Brain oedema	382 (64.8)	210 (65.0)	172 (64.7)
Complications			
IHCA	67 (11.4)	35 (10.8)	32 (12.0)
OHCA	20 (3.4)	11 (3.4)	9 (3.4)
Coma	184 (31.2)	112 (34.7)	72 (27.1)
Seizure	73 (12.4)	34 (10.5)	39 (14.7)
Epilepsy	18 (3.0)	5 (1.5)	13 (4.9)
DIC	30 (5.0)	24 (7.4)	6 (2.3)
PCCC in survivors, median (IQR)**	2 (1–3)	2 (1–2)	2 (0–3)
Evacuation of hematoma	373 (63.3)	233 (72.1)	140 (52.6)
EVD	126 (21.4)	56 (17.3)	70 (26.3)
Preoperative	xx	xx	41/70 (58.6)
Intraoperative	48 (38.1)	32/56 (57.1)	16/70 (22.8)
Postoperative	xx	xx	13/70 (18.6)
Time to EVD (days), median (IQR)	3.5 (1–47)	2 (1-29.5)	13 (1–73)
ICP monitoring	307 (52.1)	153 (47.4)	154 (57.9)
Preoperative	127 (41.3)	7/153 (4.5)	120/154 (77.9)
Intraoperative	159 (51.8)	132/153 (86.3)	27/154 (17.5)
Postoperative	21 (6.8)	14/153 (9.2)	7/154 (4.6)
Time to ICP (minutes), median (IQR)	84 (52–149)	67 (38–105)	120 (65–428)
Tracheostomy **	117 (27.5)	43(21.3)	74(33.0)
Gastrostomy**	68(15.9)	28(13.9)	40(19.8)
POFI, median (IQR)	0 (0–0)	0 (0–0)	0 (0–0)
ISS, median (IQR)	13 (9–22)	13 (9–22)	13 (9–19)
Single ICISS, median (IQR)	0.67(0.56–0.77)	0.62 (0.50–0.77)	0.68 (0.56–0.77)
Multiple ICISS, median (IQR)	0.11(0.02–0.32)	0.11 (0.02–0.31)	0.13 (0.03–0.34)

*DC* Decompressive craniectomy, *LOS* length of stay, *IHCA* Intrahospital cardiac arrest, *OHCA* Out of hospital cardiac arrest, *DIC* Disseminated intravascular coagulation, *PCCC* Pediatric Complex Chronic Conditions, *EVD* Extra ventricular drain, *ICP* Intracranial pressure, *POFI* Postoperative Organ Failure Index, *ISS* Injury Severity Score, *ICISS* International Classification Injury Severity Score, *IQR* Interquartile range, *xx* Censored data, *NA* not available.**Survivors of DC (*n* = 426).

## Supplementary Information

Below is the link to the electronic supplementary material.

Supplementary Material 1

## Data Availability

The datasets generated and/or analysed during the current study are not publicly available due to data protection regulations and is subject to the following licenses/restrictions: the original dataset can be accessed after inquiry to the Federal Bureau of Statistics of Germany. Requests to access these datasets should be directed to https://www.forschungsdatenzentrum.de/de. Data sources: RDC of the Federal Statistical Office and the Statistical Offices of the Federal States, dataset DOIs :2016: 10.21242/23141.2016.00.00.1.1.0, 2017: 10.21242/23141.2017.00.00.1.1.0, 2018: 10.21242/23141.2018.00.00.1.1.0, 2019: 10.21242/23141.2019.00.00.1.1.1, 2020: 10.21242/23141.2020.00.00.1.1.0, 2021: 10.21242/23141.2021.00.00.1.1.0, 2022: 10.21242/23141.2022.00.00.1.1.0.

## Abbreviations

AIS: Abbreviated Injury Scale
CI: Confidence Interval
DC: Decompressive craniectomy
DIC: Disseminated intravascular coagulation
EVD: Extra ventricular drain
GHD: German Hospital Dataset
ICISS: International Classification
ICP: Intracranial pressure
IHCA: Intrahospital cardiac arrest
ISS: Injury Severity Score
IQR: Interquartile range
LOS: Length of stay
OR: Odds Ratio
PCCC: Pediatric Complex Chronic Conditions
POFI: Postoperative Organ Failure Index
MV: Mechanical ventilation
OHCA: Out of hospital cardiac arrest
SRR: Survival Risk Ratio
TBI: Traumatic Brain Injury
